# Psychological burden, subjective complaints and their impact on the progression of verbal fluency in COVID-19 survivors and healthy controls: a longitudinal analysis

**DOI:** 10.1186/s40359-026-05929-w

**Published:** 2026-11-04

**Authors:** Vanessa Kauffmann, Susan Seibert, Michael Heneka, Jascha Rüsseler, Catherine N. Widmann

**Affiliations:** 1 Department of Psychology, University of Bamberg, Bamberg, Germany; 2 German Center for Neurodegenerative Diseases, Bonn, Germany; 3 Department of Neurology, University of Bonn Medical Center, Bonn, Germany; 4 Luxembourg Centre for Systems Biomedicine (LCSB), Department of Infectious Diseases and Immunology, University of Luxembourg, University of Massachusetts Medical School Worcester, MA Worcester, USA; 5 Institute of Innate Immunity, University of Bonn, Bonn, Germany; 6 Department of Neuroimmunology, Department of Parkinson’s Disease, Sleep and Movement Disorders, Center for Neurology, German Center for Neurodegenerative Diseases, University of Bonn Medical Center, Bonn, Germany

**Keywords:** COVID-19, Verbal fluency, Progression, Psychological burden, Subjective complaints

## Abstract

**Background:**

This study aimed to investigate the longitudinal change of verbal fluency after COVID-19 and potential influencing factors in a community-based sample. Within the COVIMMUNE-Clin study at the University of Bonn Medical Center, participants with a mild course of COVID-19 (*n* = 52), a severe course (*n* = 52), and healthy controls (*n* = 55) were compared. This focused secondary analysis examined verbal fluency as a distinct cognitive outcome within the broader study.

**Methods:**

Semantic and lexical verbal fluency, assessed using the Vienna Verbal Fluency Test (WIWO), as well as psychological burden measured by the ICD-10 Symptom Rating Scale (ISR) and subjective complaints assessed with the Everyday Cognition-12 (ECog-12) questionnaire, were administered at the first visit and again after 12 months. A linear mixed model was employed, incorporating Group (mild, severe and healthy controls) and Visit as fixed effects, with Subject and the Intercept as random effects. ISR and ECog-12 were each incorporated into separate models as fixed effects.

**Results:**

Group and psychological burden had an impact on semantic fluency, in a way that more severe disease and higher psychological burden were associated with lower performance. Contrary to the hypothesis, verbal fluency did not significantly change over 12 months. Across models, verbal fluency types showed distinctive patterns, which is reflected by the fact that psychological burden and subjective complaints only showed an association with semantic fluency performance. Lexical fluency showed no significant effects of these constructs and was primarily positively influenced by education.

**Conclusion:**

COVID-19 disease severity, psychological burden, and subjective complaints were associated with semantic fluency. These findings support further research on whether verbal fluency measures, in combination with self-reported symptoms, may contribute to practical assessment approaches following COVID-19.

**Trial registration:**

Specific details of the study design are included at the German Clinical Trials Registry (primary registry trial identifier: DRKS00023806; registration date: March 16, 2021, cross-referenced at World Health Organization’s International Clinical Trials Registry Platform [ICTRP]).

**Supplementary Information:**

The online version contains supplementary material available at https://doi.org/10.1186/s40359-026-05929-w.

## Background

The COVID-19 pandemic, caused by the SARS-CoV-2 virus, has significantly affected human health and daily life [1], including cognitive functioning. Various cognitive domains, like (working) memory, verbal fluency, and psychomotor speed, have shown measurable declines following COVID-19 infection [2, 3]. Research indicates that verbal fluency is among the most frequently impaired cognitive functions in post-COVID-19 patients [3–5]. Moreover, difficulties with verbal abilities can reduce an individual’s quality of life [6]. Cognitive problems, particularly in verbal fluency, can persist long after the acute phase of the infection [7, 8]. In fact, Post-COVID syndrome (PCS) encompasses a myriad of lasting symptoms, with fatigue, shortness of breath, and cognitive dysfunction being the most prevalent according to the World Health Organization (WHO) [9].

Early neuropsychological research on COVID-19-related cognitive dysfunction focused largely on possible hippocampal vulnerability and associated memory deficits, commonly assessed using verbal learning and memory tasks [10]. However, research soon also pointed to frontal-executive dysfunction, including deficits in verbal fluency [11]. Crucially, these two measures assess related but distinct cognitive processes, and findings in episodic memory cannot be assumed to generalize to verbal fluency. Beyond cognitive dysfunction, psychological factors also represent significant neuropsychological sequelae of PCS [12]. Studies show that individuals recovering from COVID-19 frequently exhibit increased rates of neuropsychiatric symptoms, including anxiety, posttraumatic stress, and depression, in the short term [13–15] and the long term [16]. Psychological burden has been generally linked to verbal fluency impairments [17] suggesting that it may contribute to the cognitive difficulties observed in COVID-19 survivors. Additionally, high psychological burden is a well-established risk factor for cognitive decline [18–20].

While cognitive and psychological challenges occur at an individual level, they are also shaped by broader factors, including the pandemic itself. Fatigue and cognitive problems following COVID-19 are not solely attributable to biological or physical health; psychological factors, such as mental health, and social determinants, including social support systems, also play crucial roles [21]. The pandemic context has also been linked to increased mental health problems, further underscoring the need to consider broader societal impacts when examining sequelae of COVID-19 [22, 23]. Emotional factors such as increased anxiety, depression, and stress during the COVID-19 pandemic may be particularly relevant in older adults. In this population, emotional state, cognitive functioning, and subjective well-being appear closely interrelated, with higher levels of depressive symptoms being associated with poorer global cognitive performance and reduced life satisfaction [24, 25]. Moreover, although social isolation predated the COVID-19 pandemic, pandemic-related restrictions substantially reduced opportunities for social interaction, which may have further contributed to cognitive decline in older adults [26, 27].

An understanding of cognitive difficulties is further complicated by inconsistent findings regarding the correlation between subjective and objective performance [5, 28]. Psychological distress may further contribute to this discrepancy, as anxiety, depression, and stress can influence both perceived and objectively measured cognitive functioning [29]. Additionally, self-report remains essential in assessing mental abilities when objective measures are unavailable or fail to detect subtle impairments [5]. This dual approach is particularly relevant in the context of PCS, where over one-third of COVID-19 survivors report high levels of subjective cognitive impairment, at least in the short- to medium term [8].

### Current explanatory approaches and gaps

Prior research has largely relied on brief, general screenings such as the 10-minute Mini-Mental State Examination (MMSE), merely assessing superficial measures of general cognitive function or self-reports [5]. Additionally, many studies have used clinical convenience samples and lacked contemporaneously recruited healthy control groups [5], making it difficult to distinguish between pandemic-related effects and direct consequences of COVID-19. These methodological limitations restrict a more comprehensive understanding of symptom development over time [30].

### Aim and hypotheses

This study aimed to characterize the progression of verbal fluency performance in two distinct cohorts of COVID-19-recovered patients—those with initially mild/asymptomatic (MLD) and severe (SEV) cases—compared to a healthy control group (HC). Participants in all groups (HC, MLD, SEV) were primarily recruited from the community and did not comprise a PCS clinical sample. At the start of the study, PCS was only speculated upon and yet to be identified.

The study spans a 12-month period between Visit 1 (V1) and Visit 3 (V3), allowing for a more in-depth analysis of long-term symptom patterns. The use of detailed verbal fluency assessments allows investigation of whether cognitive differences between groups persist, improve, or diminish over a 12-month period. Verbal fluency tasks are particularly suitable in this context because they can be seen as a proxy of general cognitive functioning and are sensitive to both language-related and executive cognitive dysfunction [31]. They may be influenced by subtle impairments associated with psychological distress and post-viral cognitive complaints [32].

However, semantic (category) and lexical (letter) verbal fluency recruit partially overlapping but distinct neural networks and are therefore examined separately. While both involve the left inferior frontal gyrus, lexical fluency relies more heavily on frontal executive regions, whereas semantic fluency additionally depends on temporal lobe structures involved in semantic processing [31, 33].

Furthermore, this study explores how COVID-19 burden, psychological distress, and subjective cognitive complaints relate to verbal fluency over time. Based on prior findings of greater cognitive impairment following more severe acute COVID-19 disease [34, 35], we expect that the severity of COVID-19 will be linked to verbal ability, with healthy controls performing better than those who had mild/asymptomatic or severe cases. At the first assessment (V1), verbal ability is anticipated to follow the pattern HC > MLD > SEV, and we expect this burden level to influence changes in performance by the final assessment (V3), with SEV participants showing the least improvement.

Psychological burden is another important factor, as high levels of distress have been associated with cognitive difficulties and reduced verbal fluency performance [36, 37]. We predict that greater psychological burden will correlate with lower verbal fluency scores across all groups, with the strongest effects observed in those who experienced severe COVID-19.

Additionally, subjective cognitive complaints—how individuals perceive their cognitive difficulties—may play a role in verbal ability over time. Although associations between subjective complaints and objective cognitive performance in post-COVID populations have been inconsistent, subjective complaints are closely linked to psychological distress and reduced well-being, both of which have been associated with poorer cognitive functioning [38, 39]. Therefore, we expect that participants reporting higher levels of subjective complaints, particularly those in the SEV group, would show less improvement in verbal fluency between V1 and V3. By examining these relationships, this study aims to provide a more comprehensive understanding of how COVID-19 and its psychological effects shape long-term cognitive outcomes.

## Method

### Setting and procedure

This research uses data from the *Long-term consequences of COVID-19 for pulmonary and neurocognitive disorders (COVIMMUNE-Clin)* study, a longitudinal prospective cohort study at the University of Bonn Medical Center (UKB). Data collection lasted from April 2021 to February 2023. The primary objective of COVIMMUNE-Clin was to compare neurocognitive and pulmonological outcomes among three distinct groups: mild/asymptomatic (MLD) and severe remitted (SEV) COVID-19-cases, and healthy controls (HC). The interval between the COVID-19 infection and the initial assessment (baseline) for the patient groups varied.

Participants underwent comprehensive evaluations at three different time points: baseline (time of recruitment), six months, and twelve months (visits 1, 2, and 3). However, this analysis focuses on neuropsychological data from the baseline and the 12-month visits (visits 1 and 3). Assessments included detailed neuropsychological testing, neurological examination, lung function tests, MRI scans, and blood sampling for neurodegenerative biomarker analysis.

All neuropsychological assessments were administered by professionally trained and certified psychological raters, as detailed in Widmann et al. [40]. The examinations were conducted using both paper-based and tablet-based formats, the latter utilizing the Vienna Testing System (VTS) software (Wiener Testsystem Version 8.15, Schuhfried, Mödling). The registered primary endpoint of COVIMMUNE-Clin was verbal episodic memory, operationalized as long-delayed verbal recall on the Auditory Verbal Learning Test (AWLT) at Visit 3. The study protocol further pre-specified exploratory analyses of group differences across other cognitive domains, including attention, language, and executive function. The present study constitutes a focused secondary analysis of verbal fluency within this broader neuropsychological assessment. The specific focus on verbal fluency was additionally supported by the clinical relevance of word-finding and verbal-retrieval complaints and the emerging literature on verbal fluency after COVID-19.

### Ethical considerations

The study was approved by the internal review board of the University Hospital Bonn (IRB Number 511/20 on 10.03.2021). Specific details of the study design are included at the German Clinical Trials Registry (primary registry trial identifier: DRKS00023806; registration date: March 16, 2021, cross-referenced at World Health Organization’s International Clinical Trials Registry Platform [ICTRP]). All participants gave their informed consent before any test was administered.

### Participants

This study aimed to include 150 participants, distributed equally among three groups: severe cases, mild/asymptomatic cases, and healthy controls. An initial over-recruitment strategy was implemented to mitigate potential dropouts and the risk of HC participants contracting COVID-19 during the study.

For the baseline data wave, a total of 159 individuals were included: HC (*n* = 55), SEV (*n* = 52), and MLD (*n* = 52). The general inclusion criteria required individuals aged 25 to 75, proficiency in German, with additional criteria specific to each group. Inclusion and exclusion criteria are detailed in Additional File 1. Various recruitment methods were employed. Two cohorts were contacted via letter: one from the COVID-19 Case Cluster Study (Heinsberg Study), comprising primarily severely affected patients, and another consisting of former in-patients of the UKB treated for COVID-19. A press release generated substantial media coverage in newspapers and on the radio. Additionally, posters of study details and contact information were placed in UKB waiting rooms, and advertisements on the UKB website and social media platforms (Facebook and Instagram managed by the UKB Communications Department). Interested participants underwent systematic telephone screening. As compensation, participants exclusively received free parking.

### Measures

#### Wiener wortflüssigkeitstest – vienna verbal fluency test

The *Vienna Verbal Fluency Test* (Wiener Wortflüssigkeitstest, WIWO), part of the Vienna Testing System (VTS) for cognitive functions in dementia [41], includes two subtests relevant to this analysis. The first subtest assesses *semantic fluency*, requiring participants to generate as many words as possible within a given category (e.g., animals). The second subtest assesses *lexical fluency*, in which participants list words starting with a specific letter (e.g., “S”) while adhering to specific rules (e.g., avoiding repetitions, numbers, and proper nouns). Each subtest has a one-minute time limit, and performance was measured by the total number of correct words produced. The WIWO was selected as it allows standardized, computerized assessment of semantic and lexical verbal fluency, cognitive domains previously reported to be affected following COVID-19 infection. The test demonstrates adequate reliability in a clinical standard sample (Cronbach’s *α* = 0.75 and 0.71) [41].

#### International statistical classification of diseases and related health symptom rating

The *ICD-10 Symptom Rating scale* (ISR) is a 29-item self-assessment questionnaire designed to evaluate level of psychiatric symptoms across multiple disorders, including depression, anxiety, obsessive-compulsion, somatoform and eating disorders, based on ICD-10 criteria [42]. Responses range from 0 (“not applicable”) to 4 (“extremely true”), with the total score reflecting overall symptomatic burden. A threshold score determines clinical relevance for each of these disorders. For this study, the ISR total score and subscales (continuous variables) serve as dependent variables. ISR demonstrates high reliability (Cronbach’s *α* = 0.87; 26).

#### Everyday cognition 12

The *Everyday Cognition 12* (ECog-12) inventory, adapted for self-assessment in the context of COVID-19, measures cognitive abilities relevant to daily activities. Patients rate their abilities in comparison to their pre-COVID-19 state, while healthy controls use the onset of the pandemic as a reference point [43]. This inventory consists of 12 items, including two that specifically relate to verbal functioning, such as changes in articulating thoughts in conversation. Responses ranged from 1 (“better or no change”) to 4 (“constantly much worse”), with an option of 5 (“I do not know”). The ECog-12 demonstrates excellent reliability (Cronbach’s alpha of 0.96; 27).

#### Reliable digit span

The *Reliable Digit Span* (RDS), an embedded performance validity test (PVT), is based on the highest number of digits correctly recalled in both forward and backward sequences from the *Wechsler Memory Scale - Revised* (WMS-R). An RDS score of 7 or lower has been proposed as a cut-off associated with suboptimal effort in neuropsychological assessment [44, 45]. PVTs are considered essential for verifying sufficient cognitive effort and a standard component of neuropsychological evaluations [46, 47]. Further details and results of the RDS are included in Additional File 2.

### Data analysis

Statistical analyses were conducted using IBM SPSS version 29. An a-priori sample size calculation was conducted using G*Power for detecting group differences including three groups and age as a covariate. Assuming a medium effect size (*f* = 0.28), *α* = 0.05, and 80% power, the analysis indicated a required sample size of 126 participants. To compensate for screening failures and attrition, the recruitment target was increased to 150 participants (50 per group). Data were checked for errors, missing values, and outliers, with detailed procedures provided in Additional File 2. To maintain sample integrity, outliers were winsorized at the 5th and 95th percentiles [48].

Descriptive analyses included chi-square tests, t-tests, AN(C)OVA, and Kruskal-Wallis tests for sociodemographic and COVID-19-related variables. Correlations were assessed using Kendall’s Tau.

Linear mixed models (LMMs) were used to analyze effects of psychological burden and subjective complaints on verbal fluency across timepoints. Semantic and lexical fluency were examined separately, with Years of Education as a fixed covariate and random effects assigned to Subject and Intercept. Comorbidities were included as covariates only when their inclusion improved model fit. Age was initially included as a covariate but was not retained in the final models because it did not contribute meaningfully to the outcomes. Model fit was evaluated using Bayesian Information Criterion (BIC), and bootstrapping (1000 samples) addressed assumption violations. Effect sizes were reported as eta squared (*η*^*2*^) or Cramer’s *V*, and partial eta squared (*η*_*p*_^*2*^) for LMMs. An alpha level of α = 0.05 was used for all analyses.

## Results

Baseline data included 159 participants, with 124 completing the third visit (HC = 42, MLD = 37, SEV = 44), corresponding to dropout rates of 23.6% (*n* = 13), 28.8% (*n* = 15), and 15.4% (*n* = 8), respectively. Dropout did not differ significantly between the COVID-19 survivor and healthy control groups (*χ*² = 2.73, *p* = .254). Participants were aged 25 to 73 years, and 52.2% were female. Most had a university or college degree (44%) and were married (56%). The SEV group was significantly older than the MLD group. However, years of education and sex distribution did not differ significantly between groups. Additional demographic and clinical details, including group comparisons, are provided in Table 1. No participants were excluded from the analysis based on performance validity criteria.

**Table 1:** Sociodemographic and clinical baseline characteristics

Characteristics	HC (*n* = 55)	MLD (*n* = 52)	SEV (*n* = 52)	Total sample (*N* = 159)	*p*	η^2^ / V
Age, years *(M*,* SD)*	47.09, 13.19	45.35, 12.24	51.25, 11.54	47.88, 12.52	0.046*	0.039
Female *(n*,* %)*	29, 52.7%	26, 50%	28, 53.8%	83, 52.2%	0.922	0.032
Education, years *(M*,* SD)*	15.83, 2.25	15.81, 2.87	15.54, 2.57	15.728, 2.36	0.781	0.003
NoC *(median*,* IQR)*	1,0–2	1,0–2	2,1–3.75	1,0–2	< 0.001***	0.101
Days between positive COVID-19 test and visit 1, *(M*,* SD)*	-	264.66, 146.821	325.5, 153.11	295.68, 152.412	0.043*	0.040
BMI (*M*, *SD*)	25.656, 4.494	26.048, 4.684	28.100, 6.158	26.584, 5.232	0.114	0.042

Note. BMI = Body Mass Index; HC = healthy controls, MLD = mild/asymptomatic, SEV = severe; NoC = Number of comorbidities; *p < .05, **p < .01, ***p < .001. Eta squared and Cramer’s *V* indicate effect sizes: small (*η*^*2*^ ≥ 0.01; *V* ≥ 0.1), moderate (*η*^*2*^ ≥ 0.06; *V* ≥ 0.3), or large (*η*^*2*^ ≥ 0.14; *V* ≥ 0.5) effects

### Neuropsychological test and questionnaire results

**Fig. 1:**
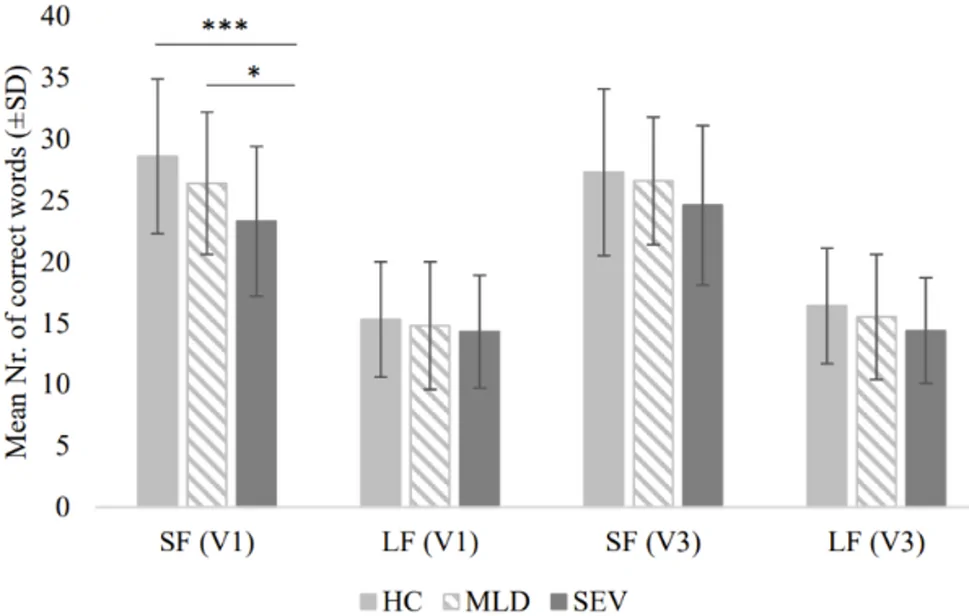
Mean scores of verbal fluency across groups at each visit. Note The mean scores are based on Bonferroni-adjusted post hoc comparisons. SD = error bars show standard deviation. SF = Semantic Fluency, LF = Lexical Fluency, HC = healthy controls, MLD = mild/asymptomatic, SEV = severe. *p < .05; **p < .01; ***p < .001

Semantic Fluency differed between groups at the first visit (*p* < .001, *η*^*2*^ = 0.116*)*. Tukey HSD post-hoc test revealed a significant difference (*p* < .001) between the HC- and the SEV-group as well as a significant difference (*p* = .025) between the MLD- and SEV-group. The average number of words per group was HC = 29, MLD = 26, SEV = 23. However, no significant group differences were observed at the third visit. No significant differences in lexical fluency were found between groups at either visit (see Fig. 1). A Kruskal-Wallis test indicated that there was a significant difference in ISR total score across groups at both visits (*p* < .001). The results for the ECog-12 total scores differed between group at both time points (*p* < .001). Post-hoc comparisons for ISR and ECog-12 revealed that the differences between all groups for all variables were significant at the *p* = .05 level. Additional File 3 provides the exact data and effect sizes. Additional File 4 provides mean scores and standard deviations of ISR, ECog-12, and verbal fluency measures at both visits, per group and for the total sample. It also reports age-adjusted ANCOVA results for the verbal fluency outcomes.

### Correlation analysis of the relevant variables

To address the relationships between psychological burden, subjective complaints, and verbal fluency, key correlations are presented in Table 2. The correlational analyses confirmed that Group was significantly associated with Semantic Fluency, ISR, and ECog-12, indicating greater symptom severity in the SEV-group. Notably, Semantic Fluency was negatively correlated with both ISR and ECog-12 at both visits, while Lexical Fluency showed no significant associations with either measure. The complete correlation matrix is provided in Additional File 5.

**Table 2:** Main Correlations (Kendall’s Tau) of Group, Semantic Fluency, ISR, and ECog-12

Variable	Group	SF V1	SF V3
ISR V1	0.340**	− 0.193**	− 0.148*
ISR V3	0.349**	− 0.148*	− 0.135*
ECog-12 V1	0.536**	− 0.289**	− 0.170*
ECog-12 V3	0.476**	− 0.249**	− 0.202*

Note. SF = Semantic Fluency, ISR = ICD-10 Symptom Rating Scale, ECog-12 = Everyday Cognition 12. *p < .05, **p < .01

### Mixed model analyses

The main outcomes of the models are presented here. Additional File 6 provides an overview of the tested models and their BIC, Additional File 7 of all fixed effects, and Additional File 8 of the significant estimated fixed effects.

#### Model 1: linking COVID-19 burden levels to verbal ability

The best-fitting model for Semantic Fluency included Visit, Group, an interaction term (Visit × Group), Education Years, and Number of Comorbidities as fixed effects along with the specified random effects subject and intercept. The model explained a substantial proportion of variance (marginal *R*^*2*^ = 0.147, conditional pseudo *R*^*2*^ =0.710), while F-statistics for Group *(p* < .001, *η*_*p*_^*2*^ *= .*111), Education Years (*p* < .001, *η*_*p*_^*2*^ *=* 0.090), and Number of Comorbidities (*p* = .004, *η*_*p*_^*2*^ *=* 0.057) were significant. Visit and the interaction term remained non-significant, indicating that while Group significantly affected Semantic Fluency, scores did not change significantly between the visits. Overall, Semantic Fluency was highest in the HC group, followed by MLD, with the SEV group showing the lowest performance across visits. Descriptively, the SEV group showed a non-significant increase in Semantic Fluency from Visit 1 (*M* = 23.29, *SD* = 6.12) to Visit 3 (*M* = 24.60, *SD* = 6.47). A separate model was fitted for Lexical Fluency. In contrast to the Semantic Fluency analysis, this model included Education Years as the only covariate, as additional covariates did not improve model fit. The model yielded a marginal *R*^*2*^ of 0.078 and a conditional pseudo *R*^*2*^ of 0.648. The significant F-statistics were those for Education Years (*p* = .003, *η*_*p*_^*2*^ *=* 0.071) and the Visit x Group interaction (*p* = .049, *η*_*p*_^*2*^ *=* 0.048*)*. Group and Visit remained non-significant, suggesting no meaningful differences in Lexical Fluency scores between groups or across visits. However, the Visit 1 × HC interaction was significant (*p* = .037), indicating group-specific differences in change between these timepoints rather than a uniform pattern across all visits.

#### Model 2: linking psychological burden to verbal ability across COVID-19 burden levels

The final linear mixed-effects model for Semantic Fluency included Education and Comorbidities as covariates. The model yielded a marginal *R*^*2*^ of 0.216 and a conditional *R*^*2*^ of 0.716. Significant fixed effects were found for Group (*p* = .022, *η*_*p*_^*2*^ *=* 0.034), ISR (*p* = .004, *η*_*p*_^*2*^ *=* 0.031), Comorbidities (*p* < .001, *η*_*p*_^*2*^ *=* 0.076), and Education Years (*p* < .001, *η*_*p*_^*2*^ *=* 0.086). ISR showed a negative association with semantic fluency, with higher psychological burden corresponding to fewer produced words.

Regarding lexical fluency, the final model included Education Years as the only covariate, with a marginal *R*^*2*^ = 0.082 and conditional *R*^*2*^ = 0.645. It demonstrated a significant fixed effect of Education Years (*p* = .003, *η*_*p*_^*2*^ *=* 0.072), while all other effects remained non-significant.

#### Model 3: linking subjective complaints to verbal ability across COVID-19 burden levels

Different model structures were selected for Semantic and Lexical Fluency based on model fit criteria. For Semantic Fluency, the best-fitting model included Visit, Group, ECog-12, their interactions, Education Years, and Number of Comorbidities as fixed effects. The model generated a marginal *R*^*2*^ of 0.210 and a conditional *R*^*2*^ of 0.724. Significant F-Statistics were found for the ECog-12 (*p* = .015, *η*_*p*_^*2*^ *=* 0.022), Education Years (*p* < .001, *η*_*p*_^*2*^ *=* 0.094), Comorbidities (*p* = .005, *η*_*p*_^*2*^ *=* 0.056), and the Group × ECog-12 interaction (*p* = .012, *η*_*p*_^*2*^ *=* 0.037).

For Lexical Fluency, the best-fitting model included Visit, Group, ECog-12, their interaction terms, and Education Years as fixed effects. The model generated a marginal *R*^*2*^ of 0.083 and a conditional *R*^*2*^ of 0.646. Significant effects were observed for Education Years (*p* = .002, *η*_*p*_^*2*^ *=* 0.075) and the Visit × Group interaction (*p* = .030, *η*_*p*_^*2*^ *=* 0.058). No significant effects were observed for ECog-12 or its interaction terms, suggesting that subjective cognitive complaints were not associated with lexical fluency performance over time.

## Discussion

This study investigated the longitudinal verbal fluency performance over 12 months in COVID-19 survivors and healthy controls, focusing on the role of psychological burden and subjective cognitive complaints. The main findings of this study indicate a dissociation between semantic and lexical verbal fluency in their associations with COVID-19 burden, psychological distress, and subjective complaints. Specifically, semantic fluency was consistently associated with group status, psychological burden, subjective complaints, and comorbidities, whereas lexical fluency showed a more restricted pattern of associations, being primarily linked to educational attainment.

In line with our first hypothesis, semantic fluency performance differed across groups, with HC outperforming both COVID-19 groups and the SEV group showing the lowest overall performance. Importantly, these effects were largely stable over the 12-month period, with limited evidence for change across visits. Although not significant, the SEV-group showed a trend toward improvement, possibly reflecting regression to the mean or pandemic effects [12]. At the same time, the longitudinal trajectories observed in the healthy control group suggest that broader pandemic-related influences may also have contributed to cognitive performance over time. A significant Visit × Group (HC) interaction emerged for lexical fluency, indicating a slight decline in HC while COVID-19 groups remained stable. However, there were no significant main effects of Visit or Group, reflecting no robust longitudinal change or between-group differences in lexical fluency. The observed patterns align with Miskowiak et al. [7], who found cognitive sequelae to be stable for one year post-hospitalization.

However, these results should be interpreted cautiously, as the absence of pre-infection data precludes conclusions regarding whether these differences reflect consequences of COVID-19 severity, pre-existing group differences, or broader contextual factors related to the pandemic experience.

Partially in line with our second hypothesis, an association was found between psychological burden and semantic fluency, with higher psychological burden being associated with lower semantic fluency performance, consistent with prior studies [18, 30]. However, no such association was observed for lexical fluency. One possible explanation is that semantic fluency relies more strongly on efficient access to stored conceptual knowledge [37], which may be particularly vulnerable to the effects of stress, and reduced cognitive efficiency commonly reported in individuals experiencing elevated psychological burden [49]. LMM analyses did not identify interaction effects, possibly due to low ISR variance (i.e., low between-subject variance and limited spread across both groups and visits, with SDs of approximately 0.5 as reported in Additional File 4).

Regarding subjective cognitive complaints, results again demonstrated a selective association with semantic fluency. Higher self-reported cognitive difficulties were linked to poorer semantic fluency performance, with this relationship differing across COVID-19 groups (Group × ECog-12 interaction). Specifically, the HC and MLD groups differed significantly from the SEV group in this relationship, indicating that the association between subjective complaints and objective performance differed across groups. This partial convergence between subjective and objective cognition is consistent with the results of some studies [38, 50], but not with others [39]. Overall, the emergence of post-COVID-19 complaints likely reflects a multifactorial construct influenced not only by objective performance. From a biopsychosocial perspective, they may arise from the relationship between perceived cognitive change, emotional distress, health status, and individual differences [29,51, 52].

The differential pattern between semantic and lexical fluency suggests partially distinct cognitive mechanisms [37, 53]. While education predicted performance in both tasks, comorbidities were selectively associated with semantic fluency, potentially reflecting a stronger reliance on semantic knowledge systems that are more sensitive to global health status and cognitive reserve. In contrast, lexical fluency appears to depend more specifically on executive retrieval strategies, which may be less affected by medical burden. Additionally, the LMMs yielded an unexpected positive association between comorbidity burden and semantic fluency performance, which may reflect selection or care-related effects in this post-COVID sample. Greater healthcare contact or access to rehabilitation or compensatory strategies among individuals with higher medical burden could partly explain this association, rather than indicating a direct beneficial effect of comorbid conditions.

Furthermore, age may influence verbal fluency performance, particularly at older ages [54]. In our analyses, age was evaluated as a potential covariate but did not improve model fit and was therefore not retained in the final models. Age-adjusted ANCOVA results are provided in Additional File 4. Although age-related effects cannot be entirely excluded, the present analyses do not indicate that age accounts for the reported group pattern.

Across models, education emerged as a robust and consistent predictor of verbal fluency performance. Notably, it was the only variable consistently associated with lexical fluency, suggesting that lexical retrieval performance in this sample was primarily explained by educational attainment rather than COVID-19-related or psychological factors. This finding is consistent with the well-established role of education as an index of cognitive reserve and verbal proficiency [55, 56].

### Practical implications

Semantic fluency is a short, cost-effective measure of verbal fluency. In this community-based sample, semantic fluency performance was associated with psychological burden and subjective cognitive complaints. The inclusion of actively recruited healthy controls is important when interpreting whether such differences may reflect COVID-19-related, pre-existing, or broader pandemic-contextual factors. Word-finding difficulties and brain fog are frequently reported by individuals recovering from COVID-19 and warrant consideration alongside objective cognitive performance and psychological burden. The present findings underscore the importance of including actively recruited control groups when attempting to distinguish disease-related effects from contextual influences. Word-finding difficulties and brain fog, frequently reported by individuals recovering from COVID-19, reflect real-life cognitive concerns [57]. Whether interventions targeting psychological burden alleviate cognitive challenges after COVID-19 requires prospective intervention studies. Evidence from stroke and cancer populations nevertheless suggests that this is a relevant question for future research [58, 59].

### Implications for future pandemics/ endemics

With the increasing frequency of global epidemics [60, 61], these findings possibly extend beyond COVID-19 to other pandemics and endemics caused by viral or other infectious diseases. Similar cognitive and psychological impairments have been observed in SARS survivors [62, 63]. These parallels suggest that the interplay between psychological burden and cognitive function could potentially constitute a common feature of post-viral syndromes.

### Interventions and future directions

Given the association between psychological distress and cognitive performance, psychoeducational or therapeutic interventions may help alleviate cognitive challenges. Research in other populations suggests that psychological interventions can improve cognitive function [58, 59, 64, ​^65^]. Similar approaches may also benefit individuals recovering from COVID-19 by addressing the psychological factors underlying cognitive deficits [66].

Future research should further investigate mechanisms linking psychological burden, depressive symptomatology, subjective complaints, and cognitive impairments, considering variables such as cogniphobia [65], fatigue, demographic influences, and potential Long COVID subtypes [67]. Methodological limitations of self-report measures, particularly for psychological burden, should be considered, including response biases and state effects, with multi-method approaches improving assessment validity [68]. Additionally, examining the cumulative effects of psychological and biological factors, including cytokine storms or amyloid-β accumulation [69], may enhance our understanding of post-COVID-19 cognitive sequelae.

### Limitations

A key limitation is the one-minute duration of verbal fluency tasks, which may have reduced sensitivity to mild deficits, as longer tasks (e.g., two minutes) are more effective in detecting subtle impairments. Additionally, only basic fluency was assessed, rather than a category-switching variant, which could have provided deeper insights into executive functions. However, the one-minute tasks were sufficiently sensitive to detect group differences and longitudinal changes in the present cohort. Nonetheless, these methodological aspects should be considered when interpreting the findings. Furthermore, our study did not include a global cognitive screening such as the MMSE or MoCA, limiting comparability with other studies. Importantly, the verbal fluency scores in the present study were analysed as raw performance measures and were not intended to establish clinical cognitive impairment. The observed group differences should therefore be interpreted as differences in verbal fluency performance rather than as evidence of clinically meaningful impairment.

In addition, a high percentage of participants in our study had higher levels of education compared to the general population, which may limit generalizability. Our participants may have increased cognitive reserve associated with higher education, potentially supporting compensatory strategies that could have attenuated observable cognitive differences in verbal fluency [56]. Additionally, although dropout did not differ between study groups, older participants were more likely to drop out by Visit 3, truncating our understanding of outcomes among older individuals. Since our sample comprised community-based volunteers rather than clinical cohorts seeking treatment for cognitive impairments following COVID-19, these results do not generalize to PCS populations. While our findings likely extend to individuals who have experienced COVID-19 in general, the extreme group comparison between mild and severe cases prevents us from confidently determining their applicability to the majority who had moderate, non-hospitalized courses. Differences in time since infection at baseline could not be modelled as a covariate because it was only available for COVID-19 participants, preventing its inclusion in between-group analyses. Furthermore, formal assessment according to established diagnostic criteria of Myalgic Encephalomyelitis / Chronic Fatigue Syndrome (ME/CFS) was not part of the study protocol, limiting conclusions regarding overlap between post-COVID cognitive symptoms and ME/CFS-related symptomatology.

## Conclusion

In conclusion, cognitive outcomes following COVID-19 as operationalized by semantic fluency performance, were consistently associated not only with disease severity, but also psychological burden across the observation period. Importantly, subjective complaints were differentially associated with objective performance across groups, highlighting that their relationship to cognition is not uniform but context-sensitive. The present pattern of results suggests that semantic fluency appears to be particularly sensitive to a combination of psychological burden and overall health status, whereas lexical fluency is comparatively stable and more strongly related to educational attainment. These findings support a multifaceted assessment of post-COVID cognitive difficulties, including consideration of broader contextual influences, and underscore the limitations of relying on a single indicator. A combined assessment approach appears advantageous for identifying appropriate intervention targets. Verbal fluency tests and self-report measures—administrable via telehealth or telephone—may serve as efficient tools for triaging individuals in need of comprehensive neuropsychological evaluations when in-person visits are not feasible. Future research targeting this possibility is warranted. Lastly, recognizing the broader impact of societal and pandemic-related stressors, even among the healthy population, is crucial for distinguishing infection-related effects from broader contextual influences.

## Supplementary Information

Below is the link to the electronic supplementary material.

Supplementary Material 1

Supplementary Material 2

Supplementary Material 3

Supplementary Material 4

Supplementary Material 5

Supplementary Material 6

Supplementary Material 7

Supplementary Material 8

## Data Availability

The datasets used and analyzed during the current study are available from the corresponding author upon reasonable request.

## Abbreviations

BMBF: German Education Ministry
COVID-19: Infectious disease caused by the Sars-CoV-2 virus
COVIMMUNE-Clin study: Long-term consequences of COVID-19 for pulmonary and neurocognitive disorders study
ECog-12: Measurement of Everyday Cognition 12 self-rating
HC: Healthy Control Group
ICD-10: International Statistical Classification of Diseases and Related Health Problems
ICTRP: World Health Organization’s International Clinical Trials Registry Platform
ISR: ICD-10 Symptom Rating
LF: Lexical Fluency
ME/CFS: Myalgic Encephalomyelitis / Chronic Fatigue Syndrome
MLD: Mild/Asymptomatic Group
MMSE: Mini-Mental State Examination
MoCA: Montreal Cognitive Assessment
MRI: Magnetic Resonance Imaging
PCS: Post-COVID Syndrome
PVT: Performance Validity Tests
RDS: Reliable Digit Span
S1: First subtest assessing Semantic Fluency, Vienna Testing System
S3: Third subtest assessing Lexical Fluency, Vienna Testing System
Sars-CoV-2: Novel coronavirus, causing COVID-19
SEV: Severe Group
SF: Semantic Fluency
UKB: = University of Bonn Medical Center
V1: Visit 1
V3: Visit 3
VF: Verbal Fluency
VTS: Vienna Testing System
WIWO: Wiener Wortflüssigkeitstest, Vienna Verbal Fluency Test
WMS-R: Wechsler Memory Scale – Revised
