# Supplementary material for: Psychological burden, subjective complaints and their impact on the progression of verbal fluency in COVID-19 survivors and healthy controls: a longitudinal analysis
Source: BMC Psychol. 2026 Nov 4;14:1597. doi: 10.1186/s40359-026-05929-w (PMC13737687; doi:10.1186/s40359-026-05929-w)
Supplement: Supplementary file 1 [file 40359_2026_5929_MOESM1_ESM.docx]

**Additional File 1**

*Inclusion Criteria for COVIMMUNE-Clin Study*

| Group | Criteria |
| --- | --- |
| Patients: Mild/Asymptomatic | No symptoms other than anosmia and/or ageusia  Able and willing to participate throughout the study |
|  |  |
| Patients: Severe | Requiring hospital stay |
|  | Able and willing to participate throughout the study |
| Healthy Control | Deny memory concerns |
|  | Must perform > -1.0 SD on the Hopkins Verbal Learning Test |
|  | No substance abuse |
|  | No known history of or current diagnosed psychiatric illness |
|  | Negative SARS-CoV-2 Rapid Antibody Test |
| Relevant for all | Written informed consent |
|  | Aged 25 to 75 years |
|  | Fluent German language abilities |

Note. Patient subjects must belong to one of the following: asymptomatic or severely affected course of COVID-19 (SARS-CoV-2 positive) infection.

*Exclusion Criteria for COVIMMUNE-Clin study*

| Group | Criteria |
| --- | --- |
| Healthy Control | Memory performance is < -1.0 SD on the Hopkins Verbal Learning |
|  | Positive SARS- CoV-2 Rapid Antibody Test at the screening visit |
| Relevant for all | Inability to give informed consent |
|  | Any condition that clearly interferes with participation in the study |
|  | Any condition that interferes with the clinical or neuropsychological study procedures |
|  | Sensory impairment that prevents or significantly interferes with neuropsychological testing |
|  | Contradiction for MRI |
|  | Severe or unstable medical condition |
|  | Current major depressive episode |
|  | Psychotic disorder, bipolar disorder, substance abuse at the present or in the past |
|  | Known neurodegenerative disorder (Alzheimer’s disease, Parkinson’s disease, Amyotrophic Lateral Sclerosis), vascular dementia, history of stroke or |
|  | History of malignant disease |
