# Supplementary material for: Psychological burden, subjective complaints and their impact on the progression of verbal fluency in COVID-19 survivors and healthy controls: a longitudinal analysis
Source: BMC Psychol. 2026 Nov 4;14:1597. doi: 10.1186/s40359-026-05929-w (PMC13737687; doi:10.1186/s40359-026-05929-w)
Supplement: Supplementary file 2 [file 40359_2026_5929_MOESM2_ESM.docx]

**Additional File 2**

*Assumption Checks for Hypothesis-Tests*

All assumptions were checked before conducting the analysis. The normal distribution for the verbal fluency and self-rating data was assessed statistically with the Shapiro-Wilk Test and graphically via the Q-Q-plot. Deviation from normal distribution was found for the WIWO Semantic Fluency severe group at the third visit (*W* = .948, *p* = .047). Despite that, due to the large sample size, the central limit theorem makes them robust against such violations (Field, 2018). Data for the Everyday Cognition Index Score and ISR score deviated from the normal distribution in every study group, except for ISR SEV at the first visit (*W* = .960, *p* = .127). The homogeneity of the data was assessed using Levene’s test. For word fluency, semantically and lexically at both points in time, variance homogeneity could be assumed based on the data. Concerning the self-rating data (ECog-12, ISR), variance homogeneity could not be presumed at both visits. ANOVAs were used to assess group differences for Lexical, as well as Semantic Fluency at both visits; due to the violation of the presumptions and the similar distribution of the data, Kruskal-Wallis tests were used to evaluate group differences concerning the ECog-12 and ISR data. The assumptions for binomial logistic regression were assessed using various diagnostic tests. The results indicated that the assumptions of linearity, independence of errors, absence of multicollinearity, and absence of influential outliers were adequately met, supporting the validity of the binomial logistic regression model. The linear mixed models showed that only Model 1(linking COVID-19 burden levels to verbal ability) for Lexical Fluency met the assumptions. Bootstrapping was employed to address violations of model assumptions for the other LMMs, whereby 1000 samples were generated.

*Missing Data Analysis*

A missing data analysis was used to assess the extent of missingness in the dataset and to determine whether missingness is related to any observed variables. Identification in % showed that 22% of the data were missing for Semantic and Lexical Fluency, ISR and ECog-12 scores for the third visit which lines up with the 35 dropouts. Additionally, ECog-12 V3 had .6% and ECog-12 V1 1.3% missing data; this was not addressed further due to the low percentages. The Missing Completely at Random (MCAR) assumption was tested using Little's MCAR test. The results indicated that the data were consistent with the MCAR assumption, *χ²* (18) = 11.029, *p* = .893. A binary dummy variable Missing Data (0 = missing, 1 = not missing) was created for the following analyses. The chi-square test of the Group and Missing Data at V3 was not significant. Hierarchical binomial logistic regression was employed to examine the predictive utility of variables in explaining missingness. In the first step, demographic variables, such as Age, Gender, Education Years, BMI and Number of Comorbidities were entered into the model to control for their effects. The binomial logistic regression model was statistically significant, *χ²* (5) = 22.042, *p* < .001. When not entered into the model, three variables are significant: Sex (*p* = .033), Age (*p* < .001) and Number of Comorbidities (*p* = .018), which aligns with the results of t-tests and chi-square test. Of the five variables entered into the regression model, one significantly predicted missing data: Age (p = .004). In the second step, relevant variables (Semantic and Lexical Fluency, ISR and ECog-12 scores) were individually added to assess their contribution to predicting missing data. This did not result in a significantly improved accuracy of the model. Missing data was not dealt with further, as with linear mixed models the available data can be used to effectively estimate the parameters (Field, 2018).

*Performance Validity Test*

The RDS was computed for every participant at the first and the third visit. Every result was over the critical cut-off score of seven. Thus, all participants were considered to have met the test criterion, indicating that their performance in the cognitive function domain is considered reliable and there is no evidence of deliberate falsification or unrealistic response behavior.
