# Supplementary material for: Psychological burden, subjective complaints and their impact on the progression of verbal fluency in COVID-19 survivors and healthy controls: a longitudinal analysis
Source: BMC Psychol. 2026 Nov 4;14:1597. doi: 10.1186/s40359-026-05929-w (PMC13737687; doi:10.1186/s40359-026-05929-w)
Supplement: Supplementary file 3 [file 40359_2026_5929_MOESM3_ESM.docx]

**Additional File 3**

*Post-hoc Comparisons for ISR and ECog-12 Total Scores at V1 and V3*

| Group comparisons | *N* | *df* | *Test Statistic* | *p* | *η²* |
| --- | --- | --- | --- | --- | --- |
| **ISR total V1** |  |  |  |  |  |
| HC/SEV | 159 | 2 | -49.102 | <.001*** | .437 |
| MLD/SEV | 159 | 2 | -36.327 | <.001 | .319 |
| **ISR total V3** | 124 |  |  |  |  |
| HC/SEV | 124 | 2 | -37.835 | <.001*** | .387 |
| MLD/SEV | 124 | 2 | -27.376 | <.001*** | .237 |
| **ECog-12 total V1** |  |  |  |  |  |
| HC/MLD | 159 | 2 | -18.944 | .010* | .204 |
| HC/SEV | 159 | 2 | -46.685 | <.001 | .504 |
| MLD/SEV | 159 | 2 | -27.740 | <.001 | .295 |
| **ECog-12 V3** |  |  |  |  |  |
| HC/SEV | 124 | 2 | -44.286 | <.001*** | .468 |
| MLD/SEV | 124 | 2 | -32.632 | <.001*** | .336 |

*Note.* HC = healthy controls, MLD = mild/asymptomatic, SEV = severe. **p* < .05, ***p* < .01, ****p* < .001. small *η²* ≥ 0.01, medium *η²* ≥ 0.06, large *η²* ≥ 0.14.
