# Supplementary material for: Psychological burden, subjective complaints and their impact on the progression of verbal fluency in COVID-19 survivors and healthy controls: a longitudinal analysis
Source: BMC Psychol. 2026 Nov 4;14:1597. doi: 10.1186/s40359-026-05929-w (PMC13737687; doi:10.1186/s40359-026-05929-w)
Supplement: Supplementary file 4 [file 40359_2026_5929_MOESM4_ESM.docx]

**Additional File 4**

*Scales, Subscales and Test for Group Differences at the First Visit*

| (Sub)scales | HC  (*n* = 55) ^a^ | | MLD  (*n* = 52) ^b^ | | SEV  (*n* = 52) | | Total sample  (*N* = 159) ^c^ | | *p* |
| --- | --- | --- | --- | --- | --- | --- | --- | --- | --- |
|  | *M* | *SD* | *M* | *SD* | *M* | *SD* | *M* | *SD* |  |
| SF | 28.60 | 6.335 | 26.44 | 5.768 | 23.29 | 6.118 | 26.16 | 6.430 | <.001*** |
| LF | 15.27 | 4.667 | 14.79 | 5.201 | 14.31 | 4.604 | 14.80 | 4.814 | .590 |
| ISR total | .38 | .270 | .51 | .378 | .99 | .650 | .62 | .528 | <.001*** |
| ISR DS | .432 | .512 | .726 | .668 | 1.587 | .906 | .906 | .860 | <.001*** |
| ISR AS | .486 | .535 | .654 | .840 | 1.183 | 1.057 | .769 | .880 | .001** |
| ISR ZS | .369 | .557 | .442 | .717 | .744 | .956 | .516 | .770 | .128 |
| ISR SS | .182 | .326 | .417 | .673 | .917 | .986 | .499 | .769 | <.001*** |
| ISR ES | .534 | .594 | .429 | .505 | .763 | .801 | .574 | .655 | .134 |
| ISR KONZ | .78 | .712 | 1.42 | 1.242 | 2.06 | 1.178 | 1.41 | 1.181 | <.001*** |
| ISR SUIZ | .00 | .000 | .02 | .139 | .13 | .345 | .05 | .219 | .003** |
| ISR SCHL | .91 | .967 | 1.12 | 1.078 | 1.75 | 1.356 | 1.25 | 1.191 | .003** |
| ISR APPE | .13 | .336 | .31 | .544 | .60 | .823 | .34 | .625 | <.001*** |
| ISR VERG | .80 | .704 | 1.37 | 1.155 | 2.15 | 1.195 | 1.43 | 1.172 | <.001*** |
| ISR FLAS | .31 | .635 | .17 | .430 | 1.00 | 1.188 | .49 | .885 | <.001*** |
| ISR DEPE | .00 | .000 | .12 | .323 | .42 | .936 | .18 | .590 | <.001*** |
| ISR DERE | .09 | .290 | .04 | .194 | .31 | .673 | .14 | .448 | .014* |
| ISR SEXU | .05 | .229 | .08 | .334 | .19 | .561 | .11 | .399 | .230 |
| ISR SEFU | .51 | .717 | .25 | .590 | .94 | 1.074 | .57 | .861 | <.001*** |
| ISR PERS | .07 | .325 | .08 | .388 | .31 | .673 | .15 | .493 | .006** |
| ISR ANPA | .18 | .475 | .27 | .598 | .71 | 1.016 | .38 | .761 | .004** |
| ECog-12 total | 1.06 | .128 | 1.33 | .484 | 1.88 | .739 | 1.42 | .614 | <.001*** |
| ECog-12 1^a, d^ | .11 | .317 | .50 | .505 | .79 | .412 | .46 | .500 | <.001*** |
| ECog-12 2 | 1.07 | .262 | 1.62 | .771 | 2.38 | .993 | 1.68 | .909 | <.001*** |
| ECog-12 3^a, d^ | 1.07 | .328 | 1.27 | .630 | 1.81 | .951 | 1.38 | .745 | <.001*** |
| ECog-12 4 | 1.04 | .270 | 1.46 | .699 | 2.15 | 1.092 | 1.54 | .884 | <.001*** |
| ECog-12 5 | 1.02 | .135 | 1.31 | .643 | 2.02 | .918 | 1.44 | .768 | <.001*** |
| ECog-12 6 | 1.09 | .554 | 1.15 | .538 | 1.69 | 1.076 | 1.31 | .803 | <.001*** |
| ECog-12 7 | 1.00 | .000 | 1.08 | .334 | 1.38 | .867 | 1.15 | .553 | <.001*** |
| ECog-12 8 | 1.05 | .229 | 1.08 | .334 | 1.42 | .750 | 1.18 | .514 | <.001*** |
| ECog-12 9^b, d^ | 1.07 | .262 | 1.22 | .577 | 1.67 | .923 | 1.32 | .687 | <.001*** |
| ECog-12 10 | 1.115 | .356 | 1.27 | .770 | 1.77 | .921 | 1.39 | .762 | <.001*** |
| ECog-12 11 | 1.02 | .135 | 1.19 | .715 | 1.67 | .985 | 1.29 | .749 | <.001*** |
| ECog-12 12 | 1.20 | .621 | 1.79 | 1.035 | 2.42 | 1.054 | 1.79 | 1.044 | <.001*** |
| ECog-12 13 | 1.04 | .189 | 1.44 | .725 | 2.12 | 1.114 | 1.52 | .885 | <.001*** |

*Note.* An explanation for the abbreviation for the ECog-12 items and ISR subscales can be found in table *Explanations of the Abbreviations for the ECog-12 Items and ISR Subscales*. ANOVA for fluency, Kruskal-Wallis for other measures. HC = healthy control, MLD = mild/asymptomatic, SEV = severe, SF = Semantic Fluency, LF = Lexical Fluency. **p* < .05, ***p* < .01, ****p* < .001.

^a^ *n* for ECog-12 total, items HC = 54, ^b^ *n* for ECog-12, items MLD = 51, ^c^ *N* for ECog-12 = 157, ^d^ *n* for items total sample = 158.

*Scales, Subscales and Test for Group Differences at the Third Visit*

| (Sub)scales | HC  (*n* = 42) ^a^ | | | | MLD  (*n* = 37) | | | | SEV  (*n* = 44) | | | | Total sample  (*N*= 124) ^b^ | | | *p* |
| --- | --- | --- | --- | --- | --- | --- | --- | --- | --- | --- | --- | --- | --- | --- | --- | --- |
|  | *M* | | *SD* | | *M* | *SD* | | | *M* | | *SD* | | *M* | | *SD* |  |
| SF | 27.33 | 6.881 | | 26.60 | | | 5.231 | 24.60 | | 6.470 | | 26.14 | | 6.329 | | --.116 |
| LF | 16.36 | 4.689 | | 15.48 | | | 5.114 | 14.43 | | 4.256 | | 15.41 | | 4.710 | | --.165 |
| ISR total | .331 | .347 | | .440 | | | .369 | .912 | | .646 | | .570 | | .542 | | <.001*** |
| ISR DS | .441 | .563 | | .605 | | | .638 | 1.386 | | .996 | | .827 | | .868 | | <.001*** |
| ISR AS | .286 | .534 | | .513 | | | .758 | 1.028 | | .896 | | .619 | | .806 | | <.001*** |
| ISR ZS | .246 | .483 | | .324 | | | .737 | .735 | | .900 | | .443 | | .756 | | --.007** |
| ISR SS | .206 | .423 | | .395 | | | .714 | .727 | | .818 | | .449 | | .704 | | <.001*** |
| ISR ES | .556 | .650 | | .491 | | | .666 | .772 | | .920 | | .613 | | .765 | | --.418 |
| ISR KONZ | .62 | .936 | | 1.18 | | | 1.159 | 2.05 | | 1.099 | | 1.30 | | 1.216 | | <.001*** |
| ISR SUIZ | .02 | .154 | | .05 | | | .226 | .18 | | .495 | | .09 | | .338 | | --.107 |
| ISR SCHL | 1.00 | 1.059 | | .82 | | | .982 | 1.80 | | 1.304 | | 1.23 | | 1.202 | | <.01** |
| ISR APPE | .26 | .627 | | .21 | | | .474 | .41 | | .897 | | .30 | | .698 | | - .674 |
| ISR VERG | .64 | .759 | | 1.13 | | | 1.018 | 2.00 | | 1.078 | | 1.27 | | 1.114 | | <.001*** |
| ISR FLAS | .21 | .606 | | .21 | | | .577 | .66 | | .914 | | .37 | | .749 | | .002** |
| ISR DEPE | .05 | .309 | | .00 | | | .000 | .39 | | .784 | | .15 | | .526 | | <.001*** |
| ISR DERE | .05 | .309 | | .08 | | | .273 | .36 | | .718 | | .17 | | .506 | | .004** |
| ISR SEXU | .07 | .261 | | .08 | | | .359 | .16 | | .479 | | .10 | | .379 | | .572 |
| ISR SEFU | .29 | .508 | | .45 | | | .828 | 1.07 | | 1.169 | | .61 | | .943 | | <.001*** |
| ISR PERS | .10 | .370 | | .13 | | | .578 | .68 | | 1.116 | | .31 | | .810 | | <.001*** |
| ISR ANPA | .19 | .594 | | .18 | | | .609 | .70 | | 1.025 | | .37 | | .811 | | <.001*** |
| ECog-12 total | 1.142 | .344 | | 1.253 | | | .463 | 1.841 | | .752 | | 1.426 | | .634 | | <.001*** |
| ECog-12 1^c,d^ | .23 | .423 | | .34 | | | .481 | .68 | | .471 | | .43 | | .497 | | <.001*** |
| ECog-12 2 | 1.27 | .549 | | 1.45 | | | .686 | 2.41 | | .972 | | 1.73 | | .915 | | <.001*** |
| ECog-12 3 | 1.07 | .264 | | 1.13 | | | .529 | 1.61 | | .754 | | 1.28 | | .608 | | <.001*** |
| ECog-12 4 | 1.20 | .511 | | 1.42 | | | .722 | 1.95 | | 1.033 | | 1.54 | | .852 | | <.001*** |
| ECog-12 5 | 1.12 | .458 | | 1.18 | | | .457 | 1.98 | | 1.000 | | 1.45 | | .802 | | <.001*** |
| ECog-12 6 | 1.10 | .300 | | 1.21 | | | .577 | 1.50 | | .976 | | 1.28 | | .705 | | .053 |
| ECog-12 7 | 1.02 | .156 | | 1.05 | | | .226 | 1.27 | | .694 | | 1.12 | | .454 | | .037* |
| ECog-12 8 | 1.02 | .156 | | 1.16 | | | .437 | 1.41 | | .622 | | 1.20 | | .478 | | <.001*** |
| ECog-12 9 ^e^ | 1.07 | .346 | | 1.24 | | | .634 | 1.67 | | 1.063 | | 1.34 | | .788 | | <.001*** |
| ECog-12 10 | 1.22 | .525 | | 1.24 | | | .590 | 1.82 | | 1.018 | | 1.44 | | .801 | | <.001*** |
| ECog-12 11 ^f^ | 1.10 | .374 | | 1.14 | | | .536 | 1.52 | | .902 | | 1.26 | | .678 | | .004** |
| ECog-12 12 | 1.24 | .699 | | 1.45 | | | .760 | 2.41 | | 1.148 | | 1.72 | | 1.035 | | <.001*** |
| ECog-12 13 ^f^ | 1.27 | .708 | | 1.32 | | | .709 | 2.14 | | 1.173 | | 1.60 | | .985 | | <.001*** |

*Note.* An explanation for the abbreviation for the ECog-12 items and ISR subscales can be found in table *Explanations of the Abbreviations for the ECog-12 Items and ISR Subscales*. ANOVA for fluency, Kruskal-Wallis for other measures. HC = healthy control, MLD = mild/asymptomatic, SEV = severe, SF = Semantic Fluency, LF = Lexical Fluency. **p* < .05, ***p* < .01, ****p* < .001.

^a^ *n* for ECog-12 total and items HC = 41, ^b^ *N* for ECog-12 total sample = 123, ^c^ *n* for ECog-12 1 HC = 40, ^d^ *n* for ECog-12 1 total sample = 122, ^e^ *n* for ECog-12 9 SEV = 43, ^f^ *n* for ECog-12 11, ECog-12 13 MLD = 37.

*Explanations of the Abbreviations for the ECog-12 Items and ISR Subscales*

| **Abbreviation** | **Full name / explanation** |
| --- | --- |
| ISR DS | Depression |
| ISR AS | Anxiety |
| ISR ZS | Compulsion |
| ISR SS | Somatization |
| ISR ES | Eating disorder |
| ISR KONZ | Concentration |
| ISR SUIZ | Suicidality |
| ISR SCHL | Sleep problems |
| ISR APPE | Loss of appetite |
| ISR VERG | Forgetfulness |
| ISR FLAS | Flashbacks |
| ISR DEPE | Depersonalization |
| ISR DERE | Derealization |
| ISR SEXU | Sexuality |
| ISR SEFU | Sexual dysfunction |
| ISR PERS | Personality change |
| ISR ANPA | Adaptation disorder |
| ECog-12 1 | Memory: concerns about memory and cognitive performance |
| ECog-12 2 | Memory: for objects |
| ECog-12 3 | Memory: for date or day of the week |
| ECog-12 4 | Language: communicating thoughts in a conversation |
| ECog-12 5 | Language: understanding oral instructions/directions |
| ECog-12 6 | Visuospatial: reading a map and giving directions |
| ECog-12 7 | Visuospatial: finding one’s way within a familiar house or building |
| ECog-12 8 | Executive planning: considering weather changes and planning accordingly |
| ECog-12 9 | Executive planning: thinking ahead |
| ECog-12 10 | Executive organization: keeping things orderly at work/home |
| ECog-12 11 | Executive organization: checking bank statements without errors |
| ECog-12 12 | Executive divided attention: doing two things at once |
| ECog-12 13 | Executive divided attention: talking while cooking/working |

*Age-Adjusted Verbal Fluency Results (ANCOVA) at the First Visit*

| Verbal Fluency | HC  (*n* = 55) | | | | MLD  (*n* = 52) | | | | SEV  (*n* = 52) | | | *p* |
| --- | --- | --- | --- | --- | --- | --- | --- | --- | --- | --- | --- | --- |
|  | *M*_a_ | | *SE* | | *M_a_* | *SE* | | | *M_a_* | | *SE* |  |
| SF | 28.64 | .819 | | 26.57 | | | .848 | 23.12 | | .852 | | <.001*** |
| LF | 15.36 | .667 | | 14.75 | | | .690 | 14.33 | | .694 | | .564 |

*Note.* HC = healthy control, MLD = mild/asymptomatic, SEV = severe, SF = Semantic Fluency, LF = Lexical Fluency. **p* < .05, ***p* < .01, ****p* < .001.

*Age-Adjusted Verbal Fluency Results (ANCOVA) at the Third Visit*

| Verbal Fluency | HC  (*n* = 42) | | | | MLD  (*n* = 38) | | | | SEV  (*n* = 44) | | | *p* |
| --- | --- | --- | --- | --- | --- | --- | --- | --- | --- | --- | --- | --- |
|  | *M*_a_ | | *SE* | | *M_a_* | *SE* | | | *M_a_* | | *SE* |  |
| SF | 27.33 | .994 | | 26.32 | | | 1.049 | 24.77 | | .975 | | .188 |
| LF | 16.35 | .737 | | 15.50 | | | .778 | 14.54 | | .723 | | .218 |

*Note.* HC = healthy control, MLD = mild/asymptomatic, SEV = severe, SF = Semantic Fluency, LF = Lexical Fluency.
