# Supplementary material for: Psychological burden, subjective complaints and their impact on the progression of verbal fluency in COVID-19 survivors and healthy controls: a longitudinal analysis
Source: BMC Psychol. 2026 Nov 4;14:1597. doi: 10.1186/s40359-026-05929-w (PMC13737687; doi:10.1186/s40359-026-05929-w)
Supplement: Supplementary file 5 [file 40359_2026_5929_MOESM5_ESM.docx]

| Variables | 1. | 2. | 3. | 4. | 5. | 6. | 7. | 8. | 9. |
| --- | --- | --- | --- | --- | --- | --- | --- | --- | --- |
| 1. Group | **—** |  |  |  |  |  |  |  |  |
| 2. SF V1 | -.267** | **—** |  |  |  |  |  |  |  |
| 3. LF V1 | -.068 | .312** | **—** |  |  |  |  |  |  |
| 4. ISR total V1 | .340** | -.193** | -.079 | **—** |  |  |  |  |  |
| 5. ECog-12 total V1 | .536** | -.289** | -.086 | .494** | **—** |  |  |  |  |
| 6. SF V3 | -.170* | .500** | .272** | -.148* | -.170* | **—** |  |  |  |
| 7. LF V3 | -.123 | .269** | .449** | -.111 | -.169* | .312** | **—** |  |  |
| 8. ISR total V3 | .349** | -.148* | -.002 | .567** | .427** | -.135* | -.091 | **—** |  |
| 9. ECog-12 total V3 | .476** | -.249** | -.056 | .388** | .657** | -.202* | -.171* | .490** | **—** |

**Additional File 5**

*Kendall’s Tau of Group, Verbal Fluency, ISR, and ECog-12*

*Note.* both-sided test; Group was coded thus: 0 = HC, 1 = MLD, 2 = SEV. HC = healthy control, MLD = mild/asymptomatic, SEV = severe, SF = Semantic Fluency; LF = Lexical Fluency. **p* < .05, ***p* < .01, ****p* < .001.
