# Supplementary material for: Psychological burden, subjective complaints and their impact on the progression of verbal fluency in COVID-19 survivors and healthy controls: a longitudinal analysis
Source: BMC Psychol. 2026 Nov 4;14:1597. doi: 10.1186/s40359-026-05929-w (PMC13737687; doi:10.1186/s40359-026-05929-w)
Supplement: Supplementary file 6 [file 40359_2026_5929_MOESM6_ESM.docx]

**Additional File 6**

*Comparison of BIC for Models with Different Covariates for Hypothesis 1*

Formularbeginn

| Dependent variable | Included covariate(s) | BIC |
| --- | --- | --- |
| SF | None | 1757.243 |
|  | Age | 1761.842 |
|  | Education Years | 1739.785 |
|  | NoC | 1738.704 |
|  | Education Years + NoC | **1721.029** |
|  |  |  |
| LF | None | 1407.880 |
|  | Age | 1410.843 |
|  | Education Years | **1400.848** |
|  | NoC | 1409.153 |
|  | Education Years + NoC | 1402.085 |

*Note*. Each model includes fixed effects for Group, Visit, as well as the interaction term Group*Visit. The model with the lowest BIC value (in bold) was selected for the analysis. Group = HC, MLD, SEV. HC = healthy control, MLD = mild/asymptomatic, SEV = severe, SF= Semantic Fluency, LF = Lexical Fluency, NoC = number of comorbidities.

*Comparison of BIC for Models with Different Covariates for Hypothesis 2*

| Dependent variable | Included covariate(s) | BIC |
| --- | --- | --- |
| SF | None | 1739.328 |
|  | Age | 1743.920 |
|  | Education Years | 1733.027 |
|  | NoC | 1717.209 |
|  | Education Years + NoC | **1700.330** |
|  |  |  |
| LF | None | 1397.820 |
|  | Age | 1400.366 |
|  | Education Years | **1390.909** |
|  | NoC | 1398.969 |
|  | Education Years + NoC | 1391.971 |

*Note*. Each model includes fixed effects for variables Group, Visit, ISR, Education Years as well as the interactions Group*Visit, Visit*ISR and Group*ISR. The model with the lowest BIC value (bold) was selected for the analysis. Group = HC, MLD, SEV. HC = healthy control, MLD = mild/asymptomatic, SEV = severe, SF = Semantic Fluency, LF = Lexical Fluency, NoC = number of comorbidities.

*Comparison of BIC for Models with Different Covariates for Hypothesis 3*

| Dependent variable | Included covariate(s) | BIC |
| --- | --- | --- |
| SF | None | 1772.194 |
|  | Age | 1776.876 |
|  | Education Years | 1754.065 |
|  | NoC | 1753.757 |
|  | Education Years + NoC | **1735.418** |
|  |  |  |
| LF | None | 1432.794 |
|  | Age | 1435.606 |
|  | Education Years | **1425.453** |
|  | NoC | 1434.026 |
|  | Education Years + NoC | 1426.631 |

*Note.* Each model includes fixed effects for variables Group, Visit, ECog-12, Education Years as well as the interactions Group*Visit, Visit*ECog-12 and Group*ECog-12. The model with the lowest BIC value (bold) was selected for the analysis. Group = HC, MLD, SEV. HC = healthy control, MLD = mild/asymptomatic, SEV = severe, SF = Semantic Fluency, LF = Lexical Fluency, NoC = number of comorbidities.
