# Supplementary material for: Psychological burden, subjective complaints and their impact on the progression of verbal fluency in COVID-19 survivors and healthy controls: a longitudinal analysis
Source: BMC Psychol. 2026 Nov 4;14:1597. doi: 10.1186/s40359-026-05929-w (PMC13737687; doi:10.1186/s40359-026-05929-w)
Supplement: Supplementary file 7 [file 40359_2026_5929_MOESM7_ESM.docx]

**Additional File 7**

*Fixed Effects for Hypothesis 1*

| Model | Variable | *F*-Statistic | *p* |
| --- | --- | --- | --- |
| SF | Intercept | *F* (1, 143.104) = 22.266 | <.001*** |
|  | Visit | *F* (1, 125.367) = .287 | .593 |
|  | Group | *F* (2, 147.075) = 9.159 | <.001*** |
|  | Education Years | *F* (1, 142.859) = 14.091 | <.001*** |
|  | NoC | *F* (1, 139.597) = 8.365 | .004** |
|  | Visit*Group | *F* (2, 125.427) = 2.566 | .081 |
|  |  |  |  |
| LF | Intercept | *F* (1, 120.000) = 10.580 | .001** |
|  | Visit | *F* (1, 121.000) = 1.143 | .287 |
|  | Group | *F* (2, 120.000) = .529 | .590 |
|  | Education Years | *F* (1, 120.000) = 9.208 | .003** |
|  | Visit*Group | *F* (2, 121.000) = 3.083 | .049 |

*Note.* SF = Semantic Fluency, LF = Lexical Fluency. Group = HC, MLD, SEV. HC = healthy control, MLD = mild/asymptomatic, SEV = severe, NoC = number of comorbidities. **p* < .05, ***p* < .01, ****p* < .001.

*Fixed Effects for Hypothesis 2*

| Model | Variable | *F*-Statistic | *p* |
| --- | --- | --- | --- |
| SF | Intercept | *F* (1, 146.132) = 27.916 | <.001*** |
|  | Visit | *F* (1, 133.546) = .000 | .995 |
|  | Group | *F* (2, 222.833) = 3.864 | .022* |
|  | Education Years | *F* (1, 141.354) = 13.264 | <.001*** |
|  | NoC | *F* (1, 142.275) = 11.663 | <.001*** |
|  | ISR | *F* (1, 266.670) = 8.558 | .004** |
|  | Group*Visit | F (2, 127.156) = 2.636 | .076 |
|  | Group*ISR | *F* (2, 266.982) = .777 | .461 |
|  | Visit*ISR | *F* (1, 133.722) = .508 | .477 |
|  |  |  |  |
| LF | Intercept | *F* (1, 121.191) = 10.210 | .002* |
|  | Visit | *F* (1, 125.655) = 1.662 | .200 |
|  | Group | *F* (2, 187.282) = .603 | .548 |
|  | Education Years | *F* (1, 117.345) = 9.069 | .003** |
|  | ISR | *F* (1, 236.834) = .010 | .921 |
|  | Group*Visit | *F* (2, 120.921) = 1.933 | .149 |
|  | Group*ISR | *F* (2, 236.683) = .426 | .654 |
|  | Visit*ISR | *F* (1, 128.341) = .696 | .406 |

*Note.* SF = Semantic Fluency, LF = Lexical Fluency. Group = HC, MLD, SEV. HC = healthy control, MLD = mild/asymptomatic, SEV = severe, NoC = number of comorbidities. **p* < .05, ***p* < .01, ****p* < .001.

*Fixed Effects for Hypothesis 3*

| Model | Variable | *F*-Statistic | *p* |
| --- | --- | --- | --- |
| SF | Intercept | *F* (1, 145.779) = 25.471 | <.001*** |
|  | Visit | *F* (1, 124.583) = .337 | .563 |
|  | Group | *F* (2, 187.355) = 1.281 | .280 |
|  | Education Years | *F* (1, 143.119) = 14.864 | <.001*** |
|  | NoC | *F* (1, 139.625) = 8.302 | .005** |
|  | ECog-12 | *F* (1, 267.248) = 6.032 | .015* |
|  | Group*Visit | *F* (2, 124.364) = 2.313 | .103 |
|  | Group*ECog-12 | *F* (2, 205.541) = 4.498 | .012* |
|  | Visit*ECog-12 | *F* (1, 169.465) = .036 | .849 |
|  |  |  |  |
| LF | Intercept | *F* (1, 120.482) = 10.178 | .002** |
|  | Visit | *F* (1, 118.472) = 1.404 | .238 |
|  | Group | *F* (2, 153.543) = .075 | .927 |
|  | Education Years | *F* (1, 118.558) = 9.551 | .002** |
|  | ECog-12 | *F* (1, 263.985) = .033 | .855 |
|  | Group*Visit | *F* (2, 118.210) = 3.618 | .030* |
|  | Group*ECog-12 | *F* (2, 198.240) = .536 | .586 |
|  | Visit*ECog-12 | *F* (1, 173.102) = 2.017 | .157 |

*Note.* SF = Semantic Fluency; LF = Lexical Fluency. Group = HC, MLD, SEV. HC = healthy control, MLD = mild/asymptomatic, SEV = severe, NoC = number of comorbidities. **p* < .05, ***p* < .01, ****p* < .001.
