# Supplementary material for: Psychological burden, subjective complaints and their impact on the progression of verbal fluency in COVID-19 survivors and healthy controls: a longitudinal analysis
Source: BMC Psychol. 2026 Nov 4;14:1597. doi: 10.1186/s40359-026-05929-w (PMC13737687; doi:10.1186/s40359-026-05929-w)
Supplement: Supplementary file 8 [file 40359_2026_5929_MOESM8_ESM.docx]

**Additional File 8**

*Estimates of Significant Fixed Effects for Hypothesis 1 SF*

|  |  |  |  |  |  |  | 95% CI | |
| --- | --- | --- | --- | --- | --- | --- | --- | --- |
| Model | Variable | Estimator | *SE* | *df* | *T* | *p* | *LL* | *UL* |
| SF | Intercept | 11.911 | 3.035 | 147.030 | 3.925 | <.001*** | 5.913 | 17.908 |
|  | Group (HC) | 3.498 | 1.256 | 220.609 | 2.786 | .006** | 1.025 | 5.973 |
|  | Education Years | .695 | .185 | 142.859 | 3.754 | <.001*** | .329 | 1.061 |
|  | NoC | .699 | .231 | 139.597 | 2.892 | .004** | .212 | 1.126 |
|  | Visit 1 * HC | 2.320 | 1.056 | 124.400 | 2.197 | .030* | .230 | 4.409 |

*Note.* SF = Semantic Fluency. Group = HC, MLD, SEV. HC = healthy control, MLD = mild/asymptomatic, SEV = severe, NoC = number of comorbidities; CI = confidence interval, *LL* = lower limit, *UL* = upper limit. Visit 3 and the SEV Group served as reference category. For reasons of clarity, only the significant estimates of fixed parameters are listed. **p* < .05, ***p* < .01, ****p* < .001.

*Estimates of Significant Fixed Effects for Hypothesis 1 LF*

|  |  |  |  |  |  |  | 95% CI | |
| --- | --- | --- | --- | --- | --- | --- | --- | --- |
| Model | Variable | Estimator | *SE* | *df* | *T* | *p* | *LL* | *UL* |
| LF | Intercept | 7.479 | 2.451 | 123.712 | 3.051 | .003** | 2.628 | 12.331 |
|  | Visit 1* HC | 1.829 | .866 | 121.000 | 2.113 | .037 | .115 | 3.543 |
|  | Education Years | .464 | .153 | 120.000 | 3.035 | .003** | .161 | .766 |

*Note.* LF = Lexical Fluency. Group = HC, MLD, SEV. HC = healthy control, MLD = mild/asymptomatic, SEV = severe, CI = confidence interval; *LL* = lower limit, *UL* = upper limit. Visit 3 and the SEV Group served as reference category. For reasons of clarity, only the significant estimates of fixed parameters are listed. **p* < .05, ***p* < .01, ****p* < .001.

*Estimates of Significant Fixed Effects for Hypothesis 2 SF*

|  |  |  |  |  |  |  | 95% CI | |
| --- | --- | --- | --- | --- | --- | --- | --- | --- |
| Model | Variable | Estimator | *SE* | *df* | *T* | *p* | *LL* | *UL* |
| SF | Intercept | 14.386 | 3.152 | 161.651 | 4.565 | <.001*** | 8.163 | 20.610 |
|  | ISR | -2.551 | 1.142 | 263.639 | -2.234 | .026* | -4.799 | -.303 |
|  | Education  Years | .663 | .182 | 141.354 | 3.642 | <.001*** | .303 | 1.023 |
|  | NoC | .792 | .232 | 142.275 | 3.415 | <.001*** | .334 | 1.251 |
|  | Visit 1*HC | 2.756 | 1.212 | 126.263 | 2.274 | .025* | .357 | 5.154 |

*Note.* SF = Semantic Fluency. Group = HC, MLD, SEV. HC = healthy control, MLD = mild/asymptomatic, SEV = severe, CI = confidence interval; *LL* = lower limit, *UL* = upper limit. Visit 3 and the SEV Group serve as reference category. For reasons of clarity, only the significant estimates of fixed parameters are listed. **p* < .05, ***p* < .01, ****p* < .001.

*Estimates of Significant Fixed Effects for Hypothesis 2 LF*

|  |  |  |  |  |  |  | 95% CI | |
| --- | --- | --- | --- | --- | --- | --- | --- | --- |
| Model | Variable | Estimator | *SE* | *df* | *T* | *p* | *LL* | *UL* |
| LF | Intercept | 6.978 | 2.621 | 135.395 | 2.662 | .009** | 1.794 | 12.162 |
|  | Education  Years | .461 | .153 | 117.345 | 3.012 | .003** | .158 | .764 |

*Note.* LF = Lexical Fluency, CI = confidence interval, *LL* = lower limit, *UL* = upper limit. For reasons of clarity, only the significant estimates of fixed parameters are listed. **p* < .05, ***p* < .01, ****p* < .001.

*Estimates of Significant Fixed Effects for Hypothesis 3 SF*

|  |  |  |  |  |  |  | 95% CI | |
| --- | --- | --- | --- | --- | --- | --- | --- | --- |
| Model | Variable | Estimator | *SE* | *df* | *T* | *p* | *LL* | *UL* |
|  | Intercept | 15.017 | 3.266 | 169.677 | 4.598 | <.001*** | 8.570 | 21.464 |
| SF | ECog-12 | -1.783 | .727 | 267.248 | -2.451 | .015* | -3.216 | -.351 |
|  | Education Years | .707 | .183 | 143.119 | 3.855 | <.001*** | .345 | 1.070 |
|  | NoC | .660 | .229 | 139.625 | 2.881 | .005** | .207 | 1.113 |
|  | HC*  ECog-12 | 1.777 | .727 | 267.248 | 2.443 | .015* | .345 | 3.210 |
|  | MLD*  ECog-12 | 1.788 | .727 | 267.247 | 2.458 | .015* | .356 | 3.221 |

*Note.* SF = Semantic Fluency. Group = HC, MLD, SEV. HC = healthy control, MLD = mild/asymptomatic, SEV = severe, NoC = number of comorbidities, CI = confidence interval, *LL* = lower limit, *UL* = upper limit. Visit 3 and the SEV Group serve as reference category. For reasons of clarity, only the significant estimates of fixed parameters are listed. **p* < .05, ***p* < .01, ****p* <.001.

*Estimates of Significant Fixed Effects for Hypothesis 3 LF*

|  |  |  |  |  |  |  | 95% CI | |
| --- | --- | --- | --- | --- | --- | --- | --- | --- |
| Model | Variable | Estimator | *SE* | *df* | *T* | *p* | *LL* | *UL* |
| LF | Intercept | 7.556 | 2.671 | 141.955 | 2.829 | .005** | 2.276 | 12.837 |
|  | Education Years | .473 | .153 | 118.558 | 3.090 | .002** | .170 | .775 |
|  | Visit 1* HC | 1.992 | .881 | 118.273 | 2.261 | .026* | .248 | 3.737 |

*Note.* LF = Lexical Fluency. Group = HC, MLD, SEV. HC = healthy control, MLD = mild/asymptomatic, SEV = severe, CI = confidence interval, *LL* = lower limit, *UL* = upper limit. Visit 3 and the SEV Group serve as reference category. For reasons of clarity, only the significant estimates of fixed parameters are listed. **p* < .05, ***p* < .01, ****p* <.001.
